# The Factors Involved in Plant–Insect–Microbe Interactions Expanded: Genome Analysis and Description of *Frigoribacterium adelgis* sp. nov.

**DOI:** 10.1111/1758-2229.70283

**Published:** 2026-01-21

**Authors:** Gustė Tamošiūnaitė, Jekaterina Havelka, Raimonda Baranauskienė, Justas Lazutka, Nomeda Kuisiene

**Affiliations:** ^1^ Department of Microbiology and Biotechnology Life Sciences Center, Institute of Biosciences, Vilnius University Vilnius Lithuania; ^2^ Department of Zoology Life Sciences Center, Institute of Biosciences, Vilnius University Vilnius Lithuania; ^3^ Department of Eukaryote Gene Engineering Life Sciences Center, Institute of Biotechnology, Vilnius University Vilnius Lithuania

**Keywords:** *Adelges* (*Aphrastasia*) *pectinatae*, adelgid, carbohydrate‐active enzymes, endophytic actinobacteria, *Frigoribacterium*, plant–insect–microbe interaction

## Abstract

Actinobacteria of the genus *Frigoribacterium* were isolated from adelgid *Adelges (Aphrastasia) pectinatae* collected from a Korean fir tree. Genomic analyses revealed that these bacteria encode a range of factors that may be involved in the interactions among *Frigoribacterium* strains, adelgids and/or conifers. Secreted carbohydrate‐active enzymes were identified in the genomes, which allow these bacteria to degrade plant polysaccharides such as cellulose, xylan, pectin and mannan, the main hemicellulose component of softwood. The degradation potential of insect cuticles was investigated, and secreted chitinases belonging to the GH18 family were predicted to be present in the genomes. However, no phenotypic chitinolytic activity was detected. The potential interactions between these bacterial strains and either plants or insects were assessed, resulting in a few high‐scoring hits. The related *Frigoribacterium* genomes were compared, revealing several unique features, such as numerous orthologous gene clusters specific to these strains and five biosynthetic gene clusters. Ten genomic islands were predicted in the genomes of the adelgid‐associated strains, which contained genes responsible for adapting to environmental changes, resisting heavy metals and expanding metabolic capabilities. We propose a new species, *Frigoribacterium adelgis*, belonging to the genus *Frigoribacterium*, based on these results.

## Graphical abstract

- Aerobic Actinobacteria belonging to the genus *Frigoribacterium* were isolated from adelgid *Adelges (Aphrastasia) pectinatae* collected from a Korean fir tree.
- Genomic analysis showed that these bacteria encode a range of factors that may be involved in the interactions between *Frigoribacterium* strains, adelgids and/or Korean fir trees.
- A few distinctive characteristics of the adelgid‐associated *Frigoribacterium* sp. strains were determined. Based on these results, a new species *Frigoribacterium adelgis* was proposed.

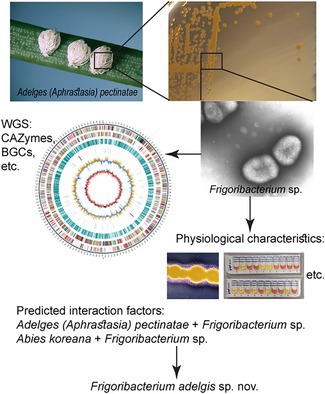

## 1 Introduction

Insect‐associated microorganisms play an important role in interactions between insects and plants. These interactions are diverse and multitrophic. Microorganisms can modulate plant metabolism and affect plant defence systems for the benefit of plants or insects. Microbes can also regulate the metabolism, development and behaviour of insects. Furthermore, secondary metabolites produced by these microbes can act as intraspecific, interspecific and mating signals in insects (Calcagnile et al. 2019). Insects can be associated with, or even transmit phytopathogenic or entomopathogenic microorganisms (Sugio et al. 2015). Primary symbionts of plant‐feeding insects ensure the health, growth and development of their hosts (Han et al. 2024). Facultative or secondary symbionts contribute to insect fitness by providing protection against natural enemies and heat stress (Oliver et al. 2010) or by determining host plant specificity (Tsuchida et al. 2004). Epiphytic and endophytic bacteria participate in plant–insect interactions. These bacteria produce phytohormones, enhance plant resistance to pests and even kill herbivorous insects (Gouda et al. 2016; Smee et al. 2021; Coolen et al. 2022). Some insects transmit plant‐beneficial endophytic bacteria (Galambos et al. 2021). However, the mechanisms by which microorganisms influence the biology of plants and insects and their interactions are largely unknown (Coolen et al. 2022).

Insects belonging to the family *Adelgidae* are a sister group of *Aphididae* and *Phylloxeridae* within *Hemiptera* that feed on host plant species by sucking phloem sap (Dancewicz et al. 2021). Adelgids are found exclusively on plants belonging to the family *Pinaceae*, and their initial generations develop on primary hosts (*Picea* spp.) following sexual reproduction and then migrate to secondary hosts belonging to the genera *Abies*, *Larix*, *Pinus*, *Tsuga* and *Pseudotsuga* (Havelka et al. 2021). Adelgid feeding results in altered solute transport, lower water availability, bud death, needle fall and decreased tree growth. Trees can completely lose their needles and desiccate within a few years (McCarty and Addesso 2019). Invasive adelgids spread rapidly and cause significant damage owing to a lack of co‐evolved plant defence mechanisms and natural enemies that can suppress the populations of these pests (McCarty and Addesso 2019; Crandall et al. 2022). Although there are some factors affecting resistance in coniferous trees, the roles of microorganisms in this process are not clearly known.

Primary endosymbionts ensure the survival of adelgids. The bacteriocytes of these insects harbour two different endosymbionts belonging to either *Gammaproteobacteria* or *Betaproteobacteria* class (Havelka et al. 2021). To the best of our knowledge, the interactions between adelgids and endosymbiotic bacteria—including microscopic studies of identity, locations, structural details and genome analysis of endosymbionts (Toenshoff et al. 2012, 2014; Michalik et al. 2013; von Dohlen et al. 2013, 2017; Dial et al. 2022; Szabó et al. 2022)—are the only focus of studies on insect–microorganism interactions. Among the species of the family *Adelgidae*, *Adelges* (*Aphrastasia*) spp. seem to be the least studied, and histological analyses have shown the presence of bacteriocytes containing bacterial symbionts (Steffan 1968), which were identified by sequencing the 16S rRNA gene fragment (Havelka et al. 2021).

In this study, we aimed to characterise culturable bacteria from adelgids *Adelges (Aphrastasia) pectinatae* collected from a Korean fir tree and to identify potential factors encoded in the genomes of these bacteria that may be involved in the interactions between bacteria, adelgids and/or their secondary host plant, the Korean fir tree. Based on the unique characteristics of these bacteria, we describe a new bacterial species, *Frigoribacterium adelgis*. To the best of our knowledge, this is the first report of isolation of *Frigoribacterium* from adelgids. This genus has rarely been isolated from other arthropods. To the best of our knowledge, *Frigoribacterium* sp. has only been isolated from ticks and nurse bees, and its role in arthropods and insects in particular remains unclear.

## 2 Experimental Procedures

### 2.1 *Adelges (Aphrastasia) pectinatae* Sample Collection

For this study, we analysed a population of *A*. (*Aphrastasia*) *pectinatae* from the Botanical Garden of Vilnius University (Kairėnai, Vilnius, Lithuania; 54.73614°N, 25.40503°E). Twigs of Korean fir (*Abies koreana*) were cut on 14 September 2023 and stored at 4°C until further analysis. A stereomicroscope (Nikon C‐PS) was used to identify first‐instar hibernating nymphs that were collected from needles and placed in sterile saline. An illustrated guide by Albrecht (2017) was used to identify the adelgid species.

### 2.2 Isolation, Genotyping and Identification of Bacterial Strains

To isolate bacteria involved in any type of bacteria–plant–insect interactions, the surfaces of the insects were not disinfected. Adelgids were aseptically homogenised until a uniform biomass was obtained. Then, 10‐fold serial dilutions of the biomass were prepared in sterile saline, and 100 μL aliquots of each dilution were plated on Luria–Bertani (LB) agar (Carl Roth GmbH + Co. KG, Germany), R2A agar, Tryptic Soy agar (TSA) (Merck Millipore, Darmstadt, Germany) and DSMZ medium No. 1021 ( *Sodalis glossinidius* medium). The latter was modified by removing serum from its composition. To isolate different microorganisms both from the surface and gut of adelgids, inoculated plates were incubated at 28°C for 48 h under both aerobic and anaerobic conditions. Cells were cultured under anaerobic conditions using Anaerocult A (Merck Millipore, Darmstadt, Germany) according to the manufacturer's instructions. Genomic DNA was extracted from fresh cultures using a GeneJET Genomic DNA Purification Kit (Thermo Fisher Scientific, Waltham, MA, USA). To exclude further analysis of the re‐isolated strains, BOX‐PCR and (GTG)_5_‐PCR genotyping experiments were performed (Wiid et al. 1994; Koeuth et al. 1995). Both genotyping experiments were performed in 50 μL of reaction mixture containing DreamTaq Green PCR Master Mix (2×) (Thermo Fisher Scientific, Waltham, MA, USA), 0.5 μM BOXA1R primer (5′‐CTA CGG CAA GGC GAC GCT GAC G‐3′) (for BOX‐PCR) or (GTG)_5_ primer (5′‐GTG GTG GTG GTG GTG‐3′) (for (GTG)_5_‐PCR), and 10 ng of bacterial genomic DNA in an Eppendorf Mastercycler EP Gradient (Eppendorf, Hamburg, Germany). PCR conditions for both genotyping experiments were as follows: initial denaturation at 95°C for 2 min followed by 29 cycles of 95°C for 1 min, 53°C for 2 min and 72°C for 3 min, with a final extension step at 72°C for 7 min. The BOX‐PCR and (GTG)_5_‐PCR genotyping profiles were analysed using electrophoresis on a 1% agarose gel.

A maximum‐likelihood 16S rRNA gene phylogenetic tree was constructed using the IQ‐TREE software (v1.6.12) with the GTR + F + I + G4 model of sequence evolution (Hoang et al. 2018). The 16S rRNA gene used for alignment was 1425 nt in size. Ultrafast bootstrap analysis of the maximum‐likelihood data, using 1000 resamplings, was performed to evaluate the validity and reliability of the tree topology.

For species identification, sequenced genomes were analysed using the JSpeciesWS server. Tetra Correlation Search against the entire genome reference database GenomesDB and ANIb calculations against all *Frigoribacterium* spp. genomes with reliable taxonomic assignments available in the NCBI Genome database at the time of writing (62 genomes, listed in Table S1) were analysed. Simultaneously, dDDH calculations for all 62 *Frigoribacterium* spp. genomes were performed using the TYGS server. It should be noted that out of three validly described species of the genus *Frigoribacterium—Frigoribacterium faeni*, *Frigoribacterium endophyticum* and *Frigoribacterium salinisoli* (Kämpfer et al. 2000; Wang et al. 2015; Kong et al. 2016)—at the time of writing, the reference genomes were available for only two of them— *F. faeni* and *F. endophyticum* (Table S1).

### 2.3 Transmission Electron Microscopy

Transmission electron microscopy was used to assess the physical properties of bacterial cells. Bacteria were cultured in DSMZ medium No. 1021 at 30°C for 48 h. Cells were washed with 10 mM Tris–HCl buffer (pH 7) and fixed for 1 h in a 4% paraformaldehyde solution. The fixed cells were washed and resuspended in 10 mM Tris–HCl buffer (pH 7). The suspension was then placed on a 400‐mesh formvar‐coated copper grid (Micro to Nano BV, Haarlem, the Netherlands). The samples were stained with a 2% aqueous uranyl acetate solution for 45 s at room temperature. The samples were examined under a Talos L120C electron microscope (Thermo Fisher Scientific, Waltham, MA, USA) operating at 120 kV.

### 2.4 Phenotypic Characterisation of the Sequenced Strains

The optimal growth temperature was determined by constructing growth curves. The ability to hydrolyse starch and Tween 80, and the effects of pH and NaCl concentration on the bacterial growth were examined as described by Krieg and Padgett (2011). Standard tests API 50 CH and API ZYM (bioMérieux SA, France) and the GEN III MicroPlate (Biolog Inc., Hayward, CA, USA) test were used for further phenotypic characterisation of the tested strains. The proteolytic characteristics of the strains were evaluated after growth on gelatin agar and plate‐count agar supplemented with powdered milk (Carl Roth GmbH + Co. KG, Germany). The chitin degradation potential was assessed as described by Hsu and Lockwood (1975) and Sutrisno et al. (2004). ‘*Paenibacillus tylopili*’ DSM 18927 (Kuisiene et al. 2008) and *Bacillus thuringiensis* wild‐type strain Bt 1.2 (both from the culture collection of the Department of Microbiology and Biotechnology, Vilnius University) were used as the positive controls, and *Escherichia coli* DH5α was used as the negative control. Fluorescent brightener 28 was added to the culture medium for better visualisation. The potential for xylan and carboxymethyl cellulose (CMC) degradation was evaluated as described by Gerasimova and Kuisiene (2012). Pectin degradation potential was tested as described by Takao et al. (2000).

Analyses of cellular fatty acids and polar lipids were carried out by DSMZ Services, Leibniz‐Institut DSMZ (Deutsche Sammlung von Mikroorganismen und Zellkulturen GmbH, Braunschweig, Germany). For analysis, strain D8 was grown on Trypticase Soy Broth Agar (DSMZ medium No. 535) at 30°C for 30 h under aerobic conditions.

### 2.5 Whole‐Genome Sequencing, Assembly and Analysis

Genome sequencing de novo was performed at Biomarker Technologies (BMK) GmbH (Münster, Germany) using Illumina NovaSeq PE150 for short‐read sequencing and Nanopore PromethION 48 for long‐read sequencing. Canu software (v1.5; Koren et al. 2017) was used to assemble the clean reads, and Racon software (v3.4.3) was used to correct the assembled sequences. Circlator software (v1.5.5; Hunt et al. 2015) was used to circularise the assembled sequences and resolve high‐quality ends. The completeness and contamination of the assemblies were estimated using CheckM software (v.1.2.3; Parks et al. 2015). The characteristics of the assembled genomes are presented in Table S2. Genome sequences were deposited in GenBank under accession numbers CP183185.1 (strain C1), CP183186.1 (strain D8) and CP183187.1 (strain Lb2).

Orthologous gene clusters were identified, annotated and analysed using the OrthoVenn3 tool (Sun et al. 2023). Protein sequences of the predicted genes were searched against the Kyoto Encyclopaedia of Genes and Genomes (KEGG) database using the BlastKOALA tool to determine the KO numbers and positions in the pathways (Kanehisa et al. 2025) and against the Pathogen–Host Interactions Database (PHI‐base v4.17) to predict proteins that may affect pathogen–host interactions (Urban et al. 2025). Carbohydrate‐active enzymes (CAZymes) were analysed, and their substrates were predicted using the dbCAN3 tool (Zheng et al. 2023). Only CAZymes predicted by at least two tools were selected for analysis. The SignalP 6.0 server (Nielsen et al. 2024) was used to predict the signal peptides of secreted CAZymes. Secreted CAZymes prediction results were visualised using the iTOL tool (v.6; Letunic and Bork 2024). The genome mining of the biosynthetic gene clusters (BGCs) was performed using the antiSMASH 7.0 pipeline (Blin et al. 2023). The IslandViewer 4 interface was used to predict genomic islands (Bertelli et al. 2017). Only genomic islands predicted by at least two prediction methods were selected for analysis. Prophage prediction was performed using the PhiSpy tool (v2.3; Akhter et al. 2012).

## 3 Results

### 3.1 Identification of the Bacterial Strains

**FIGURE 1:**
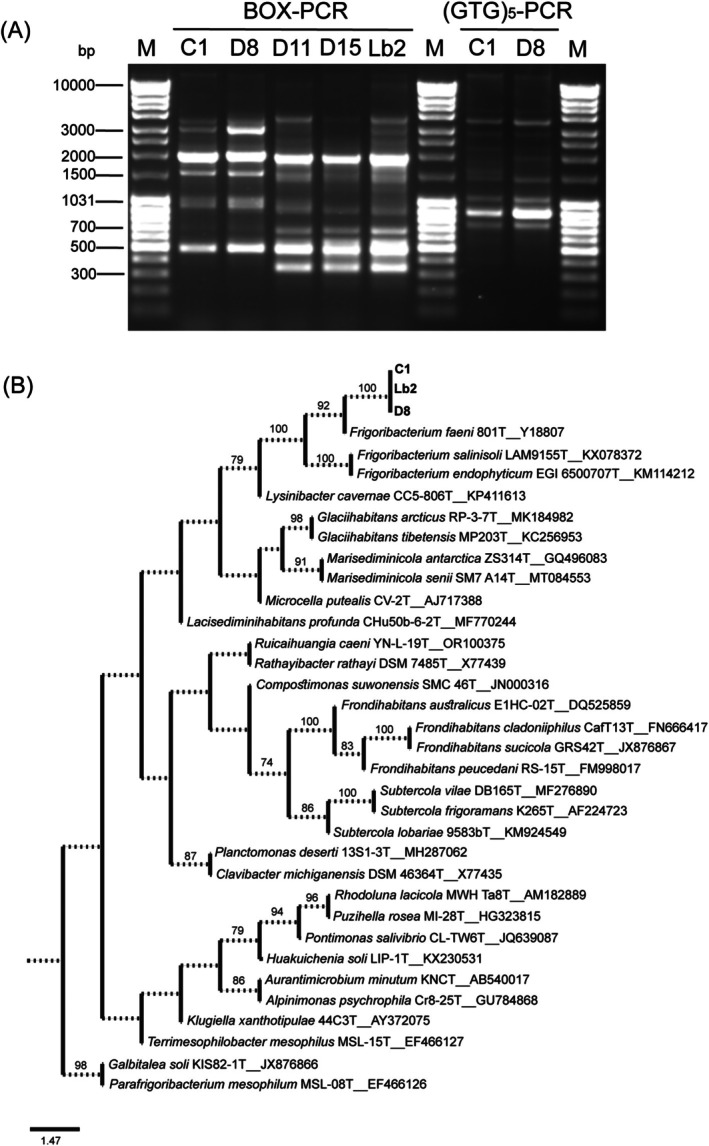
Genotyping profiles and phylogenetic position of bacterial strains isolated from the homogenised adelgids. (A) BOX‐PCR and (GTG)_5_‐PCR electrophoretic profiles. M—MassRuler DNA Ladder Mix (Thermo Fisher Scientific). (B) Phylogenetic position of the strains and the nearest neighbours from the family *Microbacteriaceae*. Scale bar, 1.47 nucleotide substitutions per site. Numbers at nodes represent ultrafast bootstrap support values based on 1000 replicates.

Our attempts to isolate microorganisms from the homogenised biomass of *A. (Aphrastasia) pectinatae* were successful only under aerobic conditions. No bacterial growth was observed under anaerobic conditions. Overall, six colony morphotypes were grown under aerobic conditions on LB agar, TSA and DSMZ medium No. 1021. The dominant morphotype on all three media was represented by yellow, round and creamy‐textured colonies, indicating that the microorganisms of this morphotype did not accidentally enter or get onto the adelgids. Therefore, five isolates of this morphotype were selected for further analysis. Isolate C1 was isolated on TSA medium; isolates D8, D11 and D15 on DSMZ medium No. 1021; and isolate Lb2 on LB agar. Genotyping experiments were performed to verify whether these isolates represented a single re‐isolated strain (Figure 1a). In the BOX‐PCR electrophoretic profiles, all isolates exhibited fragments of approximately 500 and 2000 bp, which led to the assumption that they were related. Isolates D11, D15 and Lb2 shared a 350 bp fragment that was absent from the profiles of isolates C1 and D8. The BOX‐PCR profiles of the isolates C1 and D8 were highly similar; therefore, (GTG)_5_‐PCR genotyping was performed. A few differences were identified in the 1500–2500 bp region of the (GTG)_5_‐PCR profiles of C1 and D8. The BOX‐PCR electrophoretic profiles of D11 and Lb2 were identical and differed from that of D15 in the 2000–4000 bp region. Therefore, we concluded that these five isolates represented four different strains: C1, D8, D11/Lb2 and D15. Strains C1, D8 and Lb2, isolated on different media and possibly having different genomes and/or belonging to closely related taxa, were selected for further analysis and subjected to whole‐genome sequencing.

The 16S rRNA gene sequences of all three strains were identical and most similar to those of the following validly described species belonging to the genus *Frigoribacterium* (family *Microbacteriaceae*, phylum *Actinomycetota*): *F. faeni* strain 801^T^ (sequence similarity 99.39%), *F. endophyticum* strain EGI 6500707^T^ (sequence similarity 99.06%) and *F. salinisoli* strain LAM9155^T^ (sequence similarity 98.24%). Sequence similarity with *F. salinisoli* strain LAM9155^T^ was below the threshold (98.7%) used to delineate prokaryotic species (Chun et al. 2018). The phylogenetic positions of the strains C1, D8 and Lb2 are shown in Figure 1b.

A comparison of the genomic sequences of C1, D8 and Lb2 with the reference genomes of *F. faeni* , *F. endophyticum* and *F. salinisoli* clearly showed that the strains identified in this study did not belong to these three species, as all calculated values were too low (Table 1). This indicated that strains C1, D8 and Lb2 could not be assigned to any of the validly described species of *Frigoribacterium*, representing a new species of this genus. We expanded our analysis to include all *Frigoribacterium* genomes from the Genome database of NCBI and determined that two of them, namely those of the strains *Frigoribacterium* sp. Leaf186 and *Frigoribacterium* sp. VKM Ac‐1396, could potentially be assigned to the same species based on tetra‐nucleotide and ANIb analyses; however, the dDDH values were too low (< 70.0%).

**TABLE 1:** Genome analysis–based identification of the adelgid‐associated strains.

Tested strains	Values	C1	D8	Lb2	*F. endophyticum* AS3.20^a^	*F. faeni* DSM 10309^T^	*F. faeni* NBRC 103066^T^	*F. salinisoli* JCM 30848^T^ ^b^	*F*. sp. Leaf186	*F*. sp. VKM Ac‐1396
C1	Tetra		1.0	1.0	0.93196	0.9718	0.97179	0.94369	0.99933	0.99916
	ANIb		100.0	100.0	80.85	81.5	81.56	78.92	96.23	95.11
	dDDH		100.0	100.0	23.8	25.0	24.9	22.4	68.4	61.6
D8	Tetra	1.0		1.0	0.93197	0.97181	0.9718	0.94367	0.99934	0.99916
	ANIb	100.0		100.0	80.85	81.5	81.56	78.92	96.2	95.05
	dDDH	100.0		100.0	23.8	25.0	24.9	22.4	68.4	61.6
Lb2	Tetra	1.0	1.0		0.93197	0.9718	0.9718	0.94369	0.99933	0.99916
	ANIb	100.0	100.0		80.85	81.5	81.56	78.92	96.23	95.10
	dDDH	100.0	100.0		23.8	25.0	24.9	22.4	68.4	61.6

^a^ *F*.—*Frigoribacterium*.

^b^ Analysis of this genome was done during the manuscript revision process when the reference genome of this species (acc. no. BAAGVB000000000.1) became available (26 April 2025).

### 3.2 Phenotypic Characterisation of the Adelgid‐Associated Strains

**FIGURE 2:**
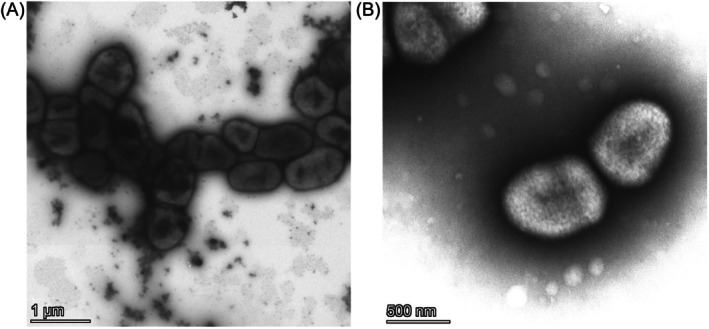
Transmission electron micrographs of strain D8 cells. (A) Cells were fixed in 4% paraformaldehyde and subsequently stained with 2% aqueous uranyl acetate. (B) Cells were stained with 2% aqueous uranyl acetate without prior fixation.

Strains C1, D8 and Lb2 (adelgid‐associated strains) were also phenotypically characterised. The cells of these strains were non‐motile irregular rods (Figure 2). The temperature range for growth was 6°C–37°C, indicating good adaptation of these strains to growth under natural conditions. Their potential for polysaccharide degradation was also investigated. The adelgid‐associated strains grew on pectin, birchwood xylan and CMC as the sole carbon sources; they were able to hydrolyse starch but not chitin. Like *E. coli* , they did not grow on the chitin‐containing medium described by Hsu and Lockwood (1975) and did not form clear hydrolysis zones on the medium described by Sutrisno et al. (2004). The strains also demonstrated proteolytic activity in media containing gelatin and powdered milk. Overall, the phenotypic properties of the strains showed a high degree of similarity (Table S3). We compared the physiological characteristics of these strains with those of three other validly published *Frigoribacterium* species, and observed that the adelgid‐associated strains differed from these species in a wide range of physiological characteristics. The most significant differences are listed in Table 2.

**TABLE 2:** Differential characteristics of the adelgid‐associated strains and the relative reference strains in the genus *Frigoribacterium*.

Characteristics	D8^T^	C1	Lb2	*F. endophyticum* JCM 30093^T^ (Wang et al. 2015)	*F. faeni* JCM 11265^T^ (Wang et al. 2015)	*F. salinisoli* LAM9155^T^ (Kong et al. 2016)
Cell width (μm)	0.55–0.6	ND^a^	ND	0.5–0.6	0.2–0.3^b^	0.3–0.7
Cell length (μm)	0.85–1.0	ND	ND	1.4–1.7	1.0–1.5^b^	0.6–1.2
Colony colour	Yellow	Yellow	Yellow	White	Yellow^b^	Yellow
Motility	−	−	−	−	+	−
Optimal growth temperature (°C)	30	30	30	25–30	20–25	25
Hydrolysis of						
Casein	+	+	+	−	−	+
Starch	+	+	+	−	−	+
Utilisation of						
Dextrin	+	+	+	−	−	−
N‐Acetyl‐β‐d‐mannosamine	+	+	+	−	−	−
l‐Fucose	+	+	+	−	−	−
Inosine	+	+	+	−	−	−
d‐Fructose 6‐phosphate	weak	−	+	−	−	−
l‐Alanine	+	+	+	−	−	−
l‐Glutamic acid	+	−	+	−	−	−
l‐Pyroglutamic acid	+	−	+	−	−	−
d‐Galacturonic acid	+	+	+	−	−	−
l‐Galactonic acid lactone	+	+	+	−	−	−
d‐Malic acid	+	+	+	−	−	−
Propionic acid	+	+	+	−	−	−
Acetic acid	+	+	+	−	−	−
Enzyme activities						
N‐Acetyl‐β‐glucosaminidase	weak	weak	weak	−	−	−
α‐Mannosidase	weak	weak	weak	−	−	−
Acid production from						
d‐Melibiose	+	+	+	−	−	−
d‐Saccharose	+	+	+	−	−	−
d‐Trehalose	+	+	+	−	−	−
Gentiobiose	+	+	+	−	−	−

*Note:* All species are positive for the following characteristics: presence of catalase, esterase lipase (C8), leucine arylamidase and naphthol‐AS‐BI‐phosphohydrolase. All species are negative for the following characteristics: H_2_S production; presence of lipase (C14), β‐glucuronidase and α‐fucosidase; utilisation of N‐acetyl‐d‐glucosamine, 3‐methyl glucose, d‐glucose 6‐phosphate, l‐serine and p‐hydroxy‐phenylacetic acid.

^a^ Not determined.

^b^ Data from Kämpfer et al. (2000).

The major polar lipids present in strain D8 were diphosphatidylglycerol and phosphatidylglycerol, similar to the other reference strains (*F. endophyticum* JCM 30093^T^, *F. faeni* DSM 10309^T^ and *F. salinisoli* LAM9155^T^). Overall, five unknown glycolipids were also detected in strain D8 (Figure S1). The major cellular fatty acids in strain D8 were anteiso‐C15:0 (38.1%), C14:0 2OH (17.0%), iso‐C16:0 (16.5%) and anteiso‐C17:0 (16.1%) (Table S4). Some differences were observed in the percentage fatty acid content among the reference strains. The cellular fatty acids C14:0 2OH and 15:0 anteiso aldehyde/15:0 anteiso DMA were identified in strain D8 and have been previously reported in the *F. faeni* type strain but not in the other two species of the *Frigoribacterium* genus. The percentages of the latter two acids differed between strains D8 and *F. faeni* DSM 10309^T^ (Table S4).

### 3.3 Genome‐Wide Analysis of Orthologous Gene Clusters

Comparative genomic analysis of orthologous gene clusters was performed to determine the distinctive genomic characteristics of the adelgid‐associated strains. For comparison, the reference genomes of *F. faeni* and *F. endophyticum* and the genomes of *Frigoribacterium* sp. strains Leaf186 and VKM Ac‐1396 were included in the analysis.

**FIGURE 3:**
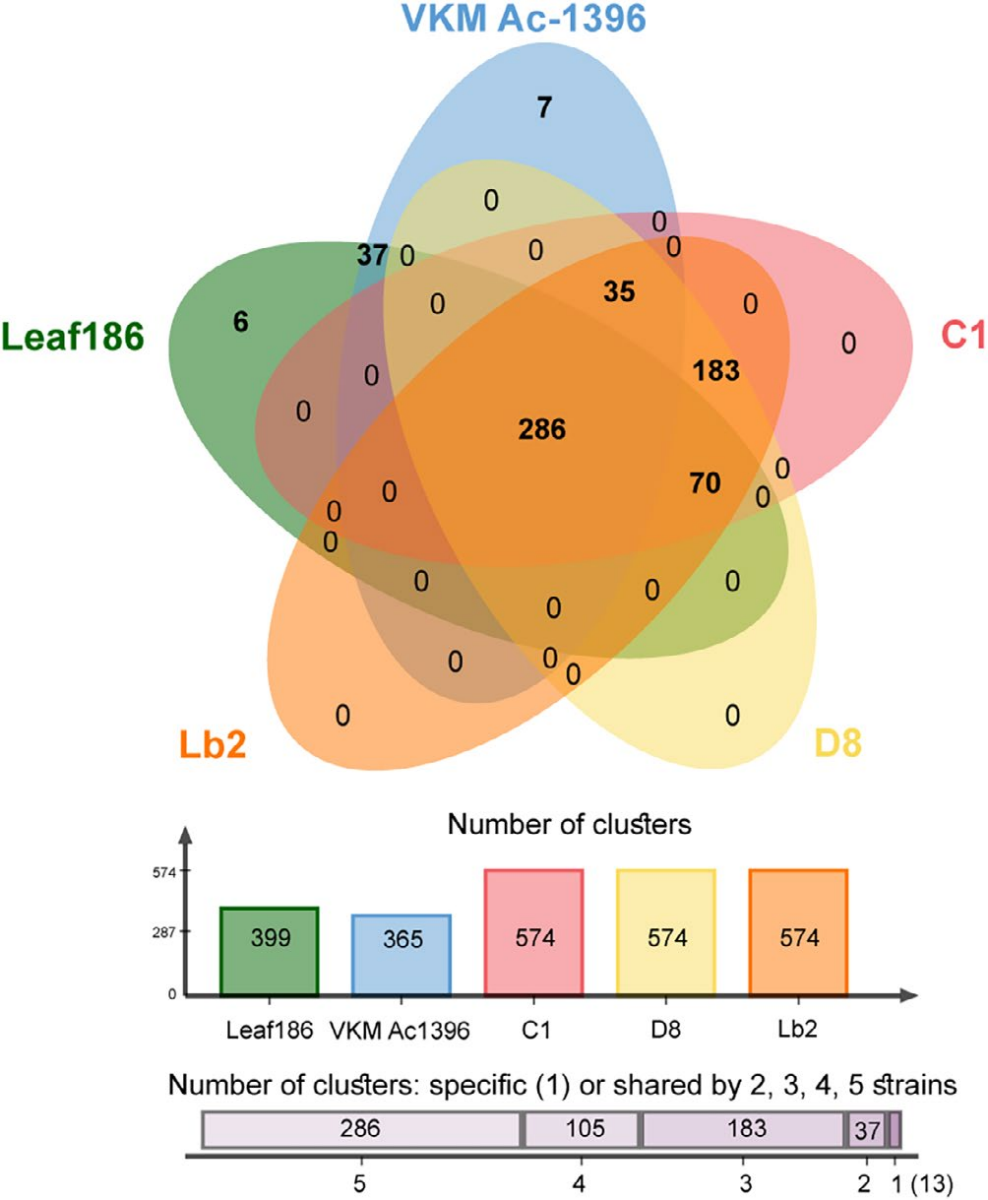
Venn diagram of orthologous clusters shared by the adelgid‐associated strains, *Frigoribacterium* sp. Leaf186 and *Frigoribacterium* sp. VKM Ac‐1396.

A total of 21,864 proteins were predicted in the genomes of the tested *Frigoribacterium* strains. They belonged to 3474 orthologous clusters, 2103 of which were single‐copy clusters. Overall, 2133 common clusters were identified among the studied genomes. The adelgid‐associated strains, *Frigoribacterium* sp. Leaf186 and *Frigoribacterium* sp. VKM Ac‐1396 shared an additional 286 clusters. Further, these strains, excluding VKM Ac‐1396, shared 70 orthologous clusters (Figure 3). *Frigoribacterium* sp. Leaf186 lacked 35 clusters shared by the adelgid‐associated strains and *Frigoribacterium* sp. VKM Ac‐1396.

We determined that 183 orthologous clusters were specific to the adelgid‐associated strains. Although some of these clusters could be attributed to molecular functions and biological processes, SwissProt hits could not be identified for most clusters, and they could not be associated with GO terms, suggesting unique roles for these proteins in adelgid‐associated strains (Table S5). Notably, half of these clusters contained proteins from the genomic islands (GIs; *see below*).

During the comparative analysis, 1211 singletons (5.54% of all proteins) were also detected. Most singletons were identified in the genomes of two *Frigoribacterium* species, *F. endophyticum* and *F. faeni* (423 and 477 proteins, respectively), whereas the number of singletons in the strains of a presumably single species, *Frigoribacterium* sp. Leaf186 and *Frigoribacterium* sp. VKM Ac‐1396, was significantly lower (184 and 126, respectively). The genomes of the adelgid‐associated strains were highly similar, and only *Frigoribacterium* sp. D8 possessed one singleton (XPP21151.1) encoded by GI_6 (*see below*). This protein was classified as hypothetical.

### 3.4 CAZymes in the Adelgid‐Associated Strains

To investigate the role of *Frigoribacterium* sp. strains in plant–insect–microbe interactions, we focused on the CAZymes encoded in the genomes of these bacteria. In every genome, a wide range of different CAZymes was predicted using at least two tools through the dbCAN3 scan. The adelgid‐associated strains and *F. endophyticum* encoded 120 CAZymes, and *Frigoribacterium* sp. VKM Ac‐1396, *Frigoribacterium* sp. Leaf186 and *F. faeni* encoded 119, 116 and 114 CAZymes, respectively. In this study, we focused on secreted CAZy proteins (Table S6) because they provide initial contact with complex polysaccharides of plant cell walls or insect exoskeletons.

In this study, we assessed whether these bacteria attack adelgids or feed on other chitin‐containing substrates (fungi) in complex biological communities on the needles of Korean fir trees. However, our cultivation‐based experiments using chitin as the sole carbon source were unsuccessful because these bacteria could not hydrolyse chitin under the experimental conditions applied in this study. Genome‐based analysis of the putative substrates revealed two genes per genome that could potentially be involved in chitin metabolism in these bacteria, namely, those of the CAZy families CBM50 and CE14; however, neither of them was predicted to be secreted (data not shown). However, bacterial chitinases mainly belong to the GH18 family and are less frequently found in the GH19 family (Oyeleye and Normi 2018). Although GH19 enzymes were not identified, GH18 enzymes were found in the genomes of all the adelgid‐associated strains and were predicted to be secreted (Table S6). This indicated that these strains have the genomic potential to feed on extracellular chitin.

*Frigoribacterium* spp. are typically considered endophytic (Tran et al. 2015; Wang et al. 2015; Singh and Dubey 2018). As potential endophytic bacteria, the adelgid‐associated strains used CMC, xylan and pectin as their sole carbon sources in our cultivation experiments. Genome‐based analysis of the putative substrates showed that secreted CAZymes of these strains could potentially utilise both α‐ and β‐mannan, β‐glucan, β‐galactan and xylan (Table S6). The genomes encode two secreted non‐orthologous GH6 endoglucanases. Although the putative substrates were not predicted, these enzymes are known to contribute to cellulose hydrolysis (Gavande and Goyal 2023). KEGG pathway analysis showed that the genomes of the adelgid‐associated strains encoded three enzymes required for the complete degradation of extracellular cellulose to glucose, K01179/EC: 3.2.1.4 (both GH6 enzymes) and K05349/EC: 3.2.1.21 (CBM32 + CBM11 + CBM11 + GH3 protein), all of which were predicted to be secreted (Figure S2; Table S6).

Secreted endo‐β‐1,4‐xylanase of the GH10 family and polysaccharide lyase of the PL9 family, well‐known for its activity against pectins, were also predicted in all three genomes of the adelgid‐associated strains. Interestingly, a few secreted GHs acting on β‐1,3 glucans (GH16, GH55 and GH81) were also detected.

In our cultivation‐based experiments, the adelgid‐associated strains hydrolysed starch, although the activity was low (data not shown). KEGG pathways analysis showed that the genomes of the adelgid‐associated strains encode all enzymes required for the complete degradation of starch (Figure S2). However, none of these were predicted to be secreted.

**FIGURE 4:**
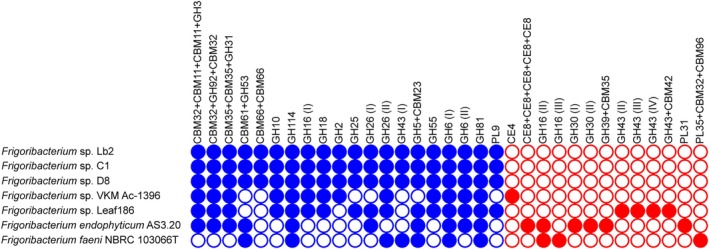
CAZy families of secreted enzymes whose genes were identified in *Frigoribacterium* sp. genomes. Blue circles indicate genes forming orthologous clusters with the genes of the adelgid‐associated strains. Genes whose orthologs were not found in the adelgid‐associated strains are marked with red circles. Filled and empty circles indicate the presence and absence, respectively, of the gene in the genome.

The predicted CAZymes secreted by the adelgid‐associated strains were compared with those in other *Frigoribacterium* genomes (Figure 4; Table S6). Our analysis revealed one protein, CBM66 + CBM66, of the adelgid‐associated strains that was not detected in the other tested genomes. Orthologous gene cluster analysis revealed that this protein was specific to these strains (Table S5). Fructans were predicted to serve as substrate for CBM66 + CBM66. Two other orthologous genes of CAZy proteins (CBM61 + GH53 and GH43) were not detected in the genomes of *Frigoribacterium* sp. Leaf186 and *Frigoribacterium* sp. VKM Ac‐1396. β‐Galactan was predicted as the substrate for CBM61 + GH53, and GH43 is well known for its arabinofuranosidase activity (Poria et al. 2020). Notably, additional non‐orthologous genes encoding secreted GH43 proteins were identified in the *Frigoribacterium* sp. Leaf186 genome (Figure 4; Table S6).

### 3.5 BGCs in the Adelgid‐Associated Strains

Overall, five different BGCs were identified in the genomes of all adelgid‐associated strains (Table 3 and Table S7). Notably, ClusterBLAST analysis revealed a few genomes with genes similar to the BGCs of the tested genomes. However, the bioactive compounds produced by these clusters remain unknown. Similarities to known clusters from the MIBiG database were very low. However, all the known clusters belonged to the class *Actinomycetes*: BGC0001666 and BGC0002690 from *Micromonospora* sp., BGC0001963 from *Catenulispora* sp. and BGC0000644 from *Dietzia* sp. None of the BGCs were predicted to be localised in the GIs.

**TABLE 3:** Biosynthetic gene clusters (BGCs) in the genomes of the adelgid‐associated strains.

BGC	Core biosynthetic genes in the cluster	Similar known gene clusters from MIBiG 3.1 (% of genes show similarity)	ClusterBLAST (100% of genes show similarity)
RiPP‐like (lactococcin 972 family bacteriocin)	Lactococcin 972 family bacteriocin	ND	*Frigoribacterium* sp. VKM Ac‐1396
β‐lactone	Pyruvate carboxylase; AMP‐dependent synthetase/ligase	BGC0001666: Microansamycins (Bossard et al. 2025)	*Frigoribacterium* sp. VKM Ac‐1396; *Frigoribacterium* sp. Leaf186
Polyketide	Type III polyketide synthase (T3PKS)	BGC0001963: Catenulisporolides (Ali et al. 2020)	*Frigoribacterium* sp. PhB24 Ga0304875_102; *Frigoribacterium* sp. Leaf254 contig 1; *Frigoribacterium* sp. VKM Ac‐2836 n1
NRPS‐independent siderophore	GNAT family N‐acetyltransferase	BGC0002690: FW0622 (Khan et al. 2020)	*Frigoribacterium* sp. VKM Ac‐1396
Terpene	Lycopene cyclase domain‐containing protein; Phytoene/squalene synthase family protein; Polyprenyl synthetase family protein	BGC0000644: Carotenoid (Stevens et al. 2021)	*Frigoribacterium* sp. VKM Ac‐1396

The BGCs of the adelgid‐associated strains were compared with those of other examined genomes. Genome mining revealed the presence of T3PKS in all isolates; however, the T3PKS of *F. endophyticum* was completely different. BGC of β‐lactone was identified only in the genomes of the adelgid‐associated strains, *Frigoribacterium* sp. Leaf186 and *Frigoribacterium* sp. VKM Ac‐1396 but not in those of *F. faeni* or *F. endophyticum*. Although the *Frigoribacterium* sp. Leaf186 genome had the highest degree of similarity to the adelgid‐associated strains (Table 1). It was found to differ significantly in terms of BGCs—the genome of this strain encodes two RiPP‐like compounds and butyrolactone, but does not encode an NRPS‐independent siderophore (data not shown).

### 3.6 GIs in the Adelgid‐Associated Strains

GIs play a role in bacterial genome diversity and contribute to bacterial fitness by conferring resistance, virulence and metabolic advantages (Munshi et al. 2025). A total of 10 GIs were predicted in the genomes of the adelgid‐associated strains using at least two prediction methods (Table S8). For strain D8, a slightly different GI_2 was predicted using only one tool; therefore, it was excluded from analysis. Two tRNAs were predicted in the GI_1 and GI_4 islands. Recombinase family proteins were predicted in GI_1, GI_3, GI_6, GI_9 and GI_10. Our results also showed that GI_4 is prophage‐derived. Tyrosine‐type recombinase/integrase was predicted in this GI.

The gene clusters of all GIs were identical in different strains, except for GI_6 (Table S8). Our analysis showed that strain D8 harbours an additional hypothetical protein on this island. Notably, all the GIs contained proteins assigned to orthologous clusters specific to the adelgid‐associated strains. In some GIs (GI_4, GI_7, GI_8, GI_9 and GI_10), these proteins constituted the absolute majority of all predicted proteins, conferring certain advantages on these strains. KEGG analysis of the GI gene content showed that KO numbers could be assigned to only one to three proteins on each island, and participation in only a few metabolic pathways could be predicted. Most GI proteins were classified as hypothetical.

To determine the possible influence of GIs on the interactions between bacterial strains and the host plants (Korean fir trees) and insects (adelgids), we focused on the prediction of encoded protein function. Our analysis revealed that some of the predicted proteins could be used by adelgid‐associated strains to adapt to the environment. A few regulators that enable bacteria to adapt to their environments and stress at the transcriptional level have been identified in four GIs—GI_3, GI_5, GI_6 and GI_8. The genes of the extracellular function sigma factors (ECF) were predicted in the GI_3 and GI_6 islands (Table S8). These sigma factors are dedicated to transcription in response to environmental changes, usually stress (Sineva et al. 2017). The MerR family of transcriptional regulators is encoded by GI_5; these regulators are involved in responses to environmental stimuli such as oxidative stress, heavy metals and antibiotics (Hobman 2007). An additional transcriptional regulator of the TetR/AcrR family was predicted in GI_8. Regulators of this family are involved in various processes, including osmotic stress, efflux pump expression and multidrug resistance (Cuthbertson and Nodwell 2013).

Proteins conferring resistance to heavy metals to the adelgid‐associated strains were also predicted in two GIs: GI_2 and GI_1. ArsR/SmtB family transcription factors (repressors) sense toxic heavy metals such as arsenic or cadmium, and their release from DNA results in the expression of resistance factors (Busenlehner et al. 2003). Heavy metal–translocating P‐type ATPase (K01533/EC: 7.2.2.9) is involved in copper extrusion from cells (Mana‐Capelli et al. 2003).

Metallopeptidases of the M23 family and serine peptidases of the S8 family were also identified in GI_1 and GI_4, respectively, enabling adelgid‐associated strains to feed more efficiently not only on plants and adelgids but also on other bacteria. It is important to note that M23 peptidase was specific to the adelgid‐associated strains in the orthologous gene cluster analysis (Table S8). S8 family subtilisin‐like peptidases are usually secreted and involved in the nutrition of bacteria, archaea and fungi, whereas M23 metallopeptidases are peptidoglycan hydrolases with a broad spectrum of activity against gram‐positive and gram‐negative bacteria (Razew et al. 2022). Proteins involved in amino acid transport and metabolism (amino acid N‐acetyltransferase in GI_1 and basic amino acid/polyamine antiporter in GI_3) complement the peptidase activity.

Efflux proteins, major facilitator superfamily proteins, dehydrogenases and oxidoreductases that have been found to be encoded in GIs could also provide additional adaptability and advantages to the adelgid‐associated strains.

### 3.7 Assessment of Potential Interactions With Plants and Adelgids

The potential interactions of adelgid‐associated bacterial strains with plants and insects were assessed using PHI‐base. Our search revealed that adelgids were not listed as hosts in this database (last checked 21 March 2025). We found only two aphid entries that were closely related to adelgids. The entomopathogenic fungi *Metarhizium robertsii* and *Beauveria bassiana* were listed as pathogens of these insects (PHI‐base entries PHI:409 and PHI:10530). Entries with conifers as hosts were also scarce. Only a few species of pine trees (*Pinus* sp.) and Chinese fir ( *Cunninghamia lanceolata* ) are listed as hosts for the fungi *Leptographium clavigerum*, *Heterobasidion annosum* and *Colletotrichum gloeosporioides* and the nematode *Bursaphelenchus xylophilus*. Therefore, to identify putative interaction factors, we conducted a PHIB‐BLAST search and limited our analysis to bacteria as pathogens and plants and insects as hosts. The top 50 hits are listed in Table S9. In terms of hosts, most hits belonged to plants, particularly eudicots. However, a few high‐scoring hits were assigned to insects, including *Drosophila melanogaster* (PHI:4712, PHI:2745 and PHI:2746; pathogenic bacteria *Pseudomonas aeruginosa* ) and moths *Galleria mellonella* (PHI:5297; pathogenic bacteria *Burkholderia cenocepacia* ). The PHI‐base entries PHI:2745 and PHI:2746 deal with trehalose biosynthesis and could be associated with both insects and plants.

To evaluate the possibility of some potential interaction factors being obtained via horizontal transfer, the PHIB‐BLAST search results were compared with the GI predictions. A single match was identified for GI_8 (Table S8). The TetR/AcrR family transcriptional regulator was found to correspond to the PHI‐base entry of *Xanthomonas campestris* (the host *Brassica oleracea* ), namely PHI:7458, but with a low score (*E* value 4.27e−7; score 47.37). The TetR/AcrR family of transcriptional regulators controls genes involved in various aspects of bacterial physiology and interacts with a wide range of small‐molecule chemicals (Wang et al. 2024).

## 4 Discussion

A growing body of literature has recognised that interactions among microorganisms, plants and insects are complex, multifaceted and multitrophic (Dove et al. 2020; Noman et al. 2020; Coolen et al. 2022). These organisms often interact positively and respond to each other's needs. However, plants also face constant threats from microbial pathogens and herbivorous insects. Similarly, microorganisms and insects face threats from each other and plants (Noman et al. 2020). Microbes play an important role in the interactions between insects and plants; however, the underlying mechanisms remain largely unknown (Coolen et al. 2022). Interactions between coniferous trees, insects and microorganisms are among the least known and studied, and are, therefore, least understood.

*Frigoribacterium* sp. have been previously isolated from airborne hay dust and air inside a museum (Kämpfer et al. 2000) and saline soils (Kong et al. 2016); however, they are usually endophytic (Wang et al. 2015; Zhou et al. 2017; Stevens et al. 2021; Kumar et al. 2025). To the best of our knowledge, there are only two reports on the isolation of *Frigoribacterium* from arthropods—one by Rudolf et al. (2009), who reported the isolation of these bacteria from surface‐sterilised ticks; and the other by Khan et al. (2020), who described the isolation of *Frigoribacterium* from the guts of nurse bees. Endophytic *Frigoribacterium* strains have been shown to be beneficial to their host plants by solubilising inorganic phosphate, producing siderophores and the phytohormones indole‐3‐acetic acid (plant auxin) and gibberellin, and synthesising 1‐aminocyclopropane‐1‐carboxylate (ACC) deaminase (Zhou et al. 2017). However, the role of Actinobacteria in arthropods remains unclear. It was hypothesised that ticks ingest them simply by chance during host‐seeking on plants and that they could survive in the tick midgut (Rudolf et al. 2009). However, nurse bee–associated bacteria appear to benefit their insect hosts by producing gluconic acid, which protects the honeybee brood from fungal pathogens (Khan et al. 2020). In the present study, three strains belonging to a single species of *Frigoribacterium* were isolated from the biomass of *A*. (*Aphrastasia*) *pectinatae* collected from a Korean fir tree. The aim of our study was to identify the partner (plant and/or adelgids) with which these bacteria interact and how this occurs.

Endophytic microorganisms are important active plant partners. They affect the growth and development of plants, biological diversity, population dynamics and functions in the ecosystem (Mamarasulov and Davranov 2024). They benefit plant growth and fitness through a variety of mechanisms: protecting plants from stresses; producing bioactive compounds, phytohormones, antimicrobial metabolites, iron chelators (siderophores) and CAZymes (chitinases); increasing nutrient availability by fixing nitrogen and solubilising inorganic phosphate; exerting insecticidal properties; stimulating the production of secondary metabolites by the host; and themselves producing metabolites similar to those of their hosts and so forth (Tiwari et al. 2023; Bossard et al. 2025; Ketehouli et al. 2025; Upadhyay and Khandelwal 2025; Zhang et al. 2025). Genome analysis of the adelgid‐associated strains showed that these bacteria do not encode the enzymes required for the biosynthesis of phytohormones (auxins and gibberellins) or ACC deaminase (data not shown), in contrast to other endophytic *Frigoribacterium* strains (Zhou et al. 2017). However, the BGC of the NRPS‐independent siderophore was predicted in the genomes of all adelgid‐associated strains. Previously, three new bacterial species have been identified in the endophytic microbiome of Korean fir tree needles, one of which, *Subtercola endophyticus*, was shown to enhance rice seedling growth by synthesising auxin and solubilising phosphate. However, its effect on the host plant, Korean fir, has not been examined and remains unknown (Jiang et al. 2022).

Bacterial endophytes attack plant cell walls by secreting CAZymes, including cellulases, xylanases, pectinases and endoglucanases (Kandel et al. 2017; Zenelt and Krawczyk 2025). Phenotypic and genomic analyses have shown that adelgid‐associated strains possess a wide range of such enzymes. These strains also encode a few secreted GHs acting on β‐1,3 glucans. Bacterial endo‐1,3‐β‐glucanases mainly belong to the GH16 family, whereas most fungal endo‐1,3‐β‐glucanases belong to GH81 (Jiang et al. 2024). In the adelgid‐associated strains, putative β‐1,3 glucanases from both the GH16 and GH81 families were predicted. Some bacterial β‐1,3 glucanases effectively inhibit the growth of phytopathogenic fungi (Jiang et al. 2024); however, the role of these enzymes in the *Frigoribacterium* strains remains undetermined. Special attention should be paid to the β‐mannanases of the GH5 and GH26 (GH26 (I) in Figure 4) families encoded in the genomes of adelgid‐associated strains. In softwood, hemicellulose is mainly present in the form of mannan, whereas in grass and hardwood, xylan is the major component of hemicellulose (Dawood and Ma 2020). This indicates that the adelgid‐associated *Frigoribacterium* strains are well adapted to reside in Korean fir trees. The assessment of potential interactions between plants and these bacterial strains also yielded a range of high‐scoring hits, in line with the endophytic role of these bacteria.

Interactions with adelgids could be of two types: beneficial or harmful. Although this is a neutral interaction, the possibility of bacteria being introduced to the adelgids as a result of it being their common habitat on the host plant or when they feed on it cannot be ruled out. We evaluated the possibility that adelgid‐associated strains produce gluconic acid in a manner analogous to *Frigoribacterium* strains isolated from nurse bees (Khan et al. 2020). Typically, gluconic acid is produced by the aerobic oxidation of glucose by glucose oxidase. The adelgid‐associated strains produced acids from glucose, galactose and mannose in our phenotypic experiments (Table S3); this activity was considered indicative of the presence of a glucose oxidase‐like enzyme (Khan et al. 2020). However, neither glucose oxidase (EC: 1.1.3.4) nor hexose oxidase (EC: 1.1.3.5) was identified in the genomes of the adelgid‐associated strains, indicating that these strains do not synthesise gluconic acid.

Bacterial chitinase production is an indicator of virulence (Oyeleye and Normi 2018). Although chitinolytic activity is usually associated with antifungal activity, chitinase production and activity are important for entomopathogenic bacteria (Singh and Mazumdar 2022). Similar to chitinase, some entomopathogenic microorganisms produce proteases that act synergistically to degrade the insect cuticle (Fang et al. 2005; Singh and Mazumdar 2022). It is important to note that chitinase overproduction enhances the virulence of the entomopathogenic fungus *B. bassiana* in aphids (Fang et al. 2005), which are closely related to adelgids. Secreted CAZymes of the family GH18 were predicted in the adelgid‐associated strains, although these strains did not use chitin as the sole carbon source. In contrast, chitin was predicted to serve as a substrate for two intracellular CAZymes of the adelgid‐associated strains, which utilised and produced acid from the chitin monomer, N‐acetyl‐d‐glucosamine, in our phenotypic experiments (Table S3). Further analysis is required to determine whether GH18 is upregulated and transcribed, especially with respect to the interactions between bacteria and adelgids. The assessment of potential interactions between insects and these bacterial strains resulted in a few high‐scoring hits, indicating that an interaction occurred.

Microbe‐produced semiochemicals play a crucial role in interorganismal communication between microorganisms, plants and insects. Secondary metabolites play key roles in this process (Ali et al. 2020; Li et al. 2022). The genomes of the adelgid‐associated strains encode five different BGCs for biosynthesising lactococcin 972 family bacteriocin, β‐lactone, polyketide (T3PKS), NRPS‐independent siderophore and terpene. β‐Lactone natural products and their derivatives have been shown to inhibit homoserine transacetylase, which is involved in the biosynthesis of methionine in many fungi, gram‐positive and some gram‐negative bacteria, but is absent in higher eukaryotes (De Pascale et al. 2011). Bacterial T3PKS produces compounds that serve as precursors for UV‐protective pigments, antibiotics and lipids, which confer antibiotic resistance, antibiotic production regulators and alternative electron carriers (Shimizu et al. 2017). Therefore, in principle, these compounds are beneficial to plants and insects because of their antifungal and antibacterial activities, and to bacteria to provide protection against competitors. However, the exact bioactive compounds encoded in the BGCs of *Frigoribacterium* strains are unknown, and their similarity to known gene clusters is low. Further research is required to determine the roles of these compounds in the adelgid‐associated strains. Our results showed that the BGCs of the adelgid‐associated strains should be considered species‐specific, as they were not encoded in the GIs and thus could not be obtained through horizontal gene transfer.

Genomic analysis of the adelgid‐associated strains showed that all the strains belonged to a single species of the genus *Frigoribacterium*. Of all *Frigoribacterium* strains examined, only strains C1, D8 and Lb2 were isolated from insects that feed on conifer needles. These strains have been isolated from the surface and/or gut of adelgids and are not primary symbionts. Previous next‐generation sequencing–based analyses of primary symbionts performed in our laboratory revealed *Sodalis* spp., but not *Frigoribacterium* (Kuisiene and Havelka, unpublished results). *Frigoribacterium* sp. 16S rRNA gene sequences were also identified in samples collected from hemlock trees affected by the adelgid *Adelges* (*Annandina*) *tsugae*; however, it should be noted that representatives of neither *Microbacteriaceae* nor *Frigoribacterium* were listed among the most abundant taxa in the samples, including needles (Dove et al. 2020). Notably, another strain from our study, *Frigoribacterium* sp. VKM Ac‐1396, was isolated from the leaf gall of the grass *Festuca rubra* induced by the plant‐parasitic nematode *Anguina graminis* (Tarlachkov et al. 2021). This indicates that out of the five strains that potentially belong to a single species, only *Frigoribacterium* sp. Leaf186 was isolated from the leaves of a seemingly healthy *Arabidopsis thaliana* plant (Bai et al. 2015).

A few distinct characteristics of the adelgid‐associated strains were determined. Comparison with relatives revealed numerous orthologous gene clusters unique to the three strains (Table S5). Hypothetical proteins that most likely play specific roles in the biology of these bacteria were identified in most of these unique clusters. Half of these clusters were found in GIs, which are usually thought to be acquired through horizontal gene transfer and confer beneficial traits to the recipient. Among the GI proteins with a predicted function, at least some could serve in adaptation to the environment and expand the metabolic capabilities of the adelgid‐associated strains.

The adelgid‐associated strains had one more unique characteristic: their genomes encoded the CBM66 + CBM66 protein, which was absent in the other genomes studied. According to the CAZy database (https://www.cazy.org/CBM66.html), most CBM66 modules are located in the GH32 enzymes that target fructans. Fructans are not synthesised by gymnosperms but can be produced by bacteria (Morvan‐Bertrand et al. 2023). For example, endophytic bacteria can synthesise fructan inulin and produce fructan‐degrading enzymes (Khamwan et al. 2022). However, fructan biosynthesis was not predicted in the genomes of the adelgid‐associated strains (data not shown), hydrolases of the GH32 family were not detected, and inulin was not utilised in phenotypic tests (Table S3). Some CBM66 members can be linked to a range of other CAZymes, but not to GH32s; these other enzymes are mainly hydrolases and lyases, which are associated with plant cell wall degradation. We hypothesised that this module is important for bacterial life in conifers; however, further research is required to confirm this hypothesis.

## 5 Conclusion

In the present study, three strains belonging to a single species of the *Frigoribacterium* genus were isolated from the biomass of *A*. (*Aphrastasia*) *pectinatae* collected from a Korean fir tree. To the best of our knowledge, this is the first study to isolate *Frigoribacterium* strains from adelgids. Genomic analysis revealed that these bacteria encode a range of factors that may be involved in the interactions between *Frigoribacterium* strains, adelgids and/or Korean fir trees. *Frigoribacterium* sp. strains exhibited several unique features, including orthologous gene clusters, CAZymes and BGCs, indicating their potential applications in plant protection and/or biocontrol against adelgids.

Based on the results of this study, we propose a new species, *F. adelgis* sp. nov.

Adelgid‐associated strains have been deposited in two microorganism culture collections (DSMZ (Germany) and BCCM/LMG (Belgium)) under the following accession numbers: D8^T^ = DSM 119719^T^ = LMG 34012^T^; C1 = DSM 119721 = LMG 34013; Lb2 = DSM 119720 = LMG 34014. The phenotypic characteristics of the new species are summarised in Tables S3 and S4.

### Description of *F. adelgis* sp. nov.

a. dell'gis. N.L. gen. n. *adelgis* of the insect *Adelges (Aphrastasia) pectinatae* from the biomass of which the organism was isolated.

Cells are aerobic, gram‐positive, non‐spore forming, non‐motile, irregular rods (0.55–0.6 μm in width and 0.85–1.0 μm in length) and produce yellow, round, creamy‐textured colonies. Growth occurs at 6°C–37°C (optimum 30°C). The pH range for growth is 6–10 (optimum pH 6). Growth occurs in the presence of 0%–7% NaCl (optimum 0%–1%). It is positive for catalase activity and utilises pectin, xylan and carboxymethyl cellulose as sole carbon sources. It hydrolyses starch (weakly), but not chitin. The DNA G + C content, as determined from the genome sequence of the type strain, is 71.5%.

The type strain is D8^T^ (=DSM 119719^T^ = LMG 34012^T^), which was isolated from the homogenised biomass of *Adelges (Aphrastasia) pectinatae*, collected from the Korean fir *A. koreana* in the Botanical Garden of Vilnius University (54.73614°N, 25.40503°E). The NCBI accession number for the complete genome sequence of the type strain is CP183186.1.

## Author Contributions

**Jekaterina Havelka:** conceptualization, methodology, writing – review and editing, resources, investigation, supervision. **Nomeda Kuisiene:** conceptualization, methodology, writing – original draft, data curation, formal analysis, writing – review and editing, project administration, supervision. **Raimonda Baranauskienė:** methodology, investigation, validation, visualization, writing – review and editing. **Gustė Tamošiūnaitė:** investigation, formal analysis, validation, writing – review and editing. **Justas Lazutka:** methodology, resources, investigation, validation, visualization, writing – review and editing.

## Conflicts of Interest

The authors declare no conflicts of interest.

## Supporting information


**TABLE S1:** Genomes of *Frigoribacterium* used in this study.
**Table S2:** The characteristics of the assembled genomes.
**Table S3:** Phenotypic characteristics of the adelgid‐associated strains.
**Table S4:** Cellular fatty acid composition (> 0.5%) of the adelgid‐associated strain D8 and its relative reference strains in the genus *Frigoribacterium*. Values are percentages of total fatty acids. ND, not detected.
**Table S5:** The adelgid‐associated strains–specific orthologous gene clusters. Clusters localised in genomic islands are in bold.
**Table S6:** Secreted carbohydrate‐active enzymes (CAZymes) predicted in *Frigoribacterium* genomes. Orthologous genes specific to the adelgid‐associated strains are in bold.
**Table S7:** Genomic location of biosynthetic gene clusters (BGCs).
**Table S8:** Characteristics of genomic islands. Orthologous proteins that were specific to the adelgid‐associated strains are in bold text.
**Table S9:** Top 50 PHIB‐BLAST search results.

**FIGURE S1:** The polar lipids of the *Frigoribacterium* sp. strain D8. DPG, diphosphatidylglycerol; GL, glycolipid; PG, phosphatidylglycerol.
**Figure S2:** Starch and cellulose metabolism in the *Frigoribacterium* sp. strain D8. Predicted proteins are marked with green rectangles. Conversion of cellulose to d‐glucose: EC 3.2.1.4.—endoglucanase; EC 3.2.1.21.—1,4‐β‐d‐glucan glucohydrolase/cellobiose glucohydrolase. Conversion of starch to d‐glucose: EC 2.4.1.64—α,α‐trehalose: orthophosphate β‐d‐glucosyltransferase; EC 3.2.1.1—α‐amylase/maltodextrin maltohydrolase; EC 3.2.1.10—dextrin 6‐α‐d‐glucanohydrolase; EC 3.2.1.133—maltogenic α‐amylase; EC 3.2.1.141–4‐α‐d‐[(1‐>4)‐α‐d‐glucano]trehalose glucanohydrolase; EC 3.2.1.20—maltose glucohydrolase; EC 3.2.1.68—isoamylase; EC 5.4.99.15—maltodextrin 1‐α‐d‐glucosylmutase; EC 5.4.99.16—maltose α‐d‐glucosylmutase.

## Data Availability

The GenBank accession numbers of de novo sequenced genomes of *Frigoribacterium* sp. strains C1, D8^T^ and Lb2 are listed in Table S2. The numbers of the strains in the microorganism culture collections are as follows: D8^T^ = DSM 119719^T^ = LMG 34012^T^; C1 = DSM 119721 = LMG 34013; Lb2 = DSM 119720 = LMG 34014. Tables S1–S9 and Figures S1 and S2 can be accessed using the following DOI: https://doi.org/10.5281/zenodo.17434754.
