# Supplementary material for: The Factors Involved in Plant–Insect–Microbe Interactions Expanded: Genome Analysis and Description of Frigoribacterium adelgis sp. nov
Source: Environ Microbiol Rep. 2026 Jan 21;18(1):e70283. doi: 10.1111/1758-2229.70283 (PMC12823222; doi:10.1111/1758-2229.70283)
Supplement: Supplementary file 2 — FIGURE S1: The polar lipids of the Frigoribacterium sp. strain D8. DPG, diphosphatidylglycerol; GL, glycolipid; PG, phosphatidylglycerol. Figure S2: Starch and cellulose metabolism in the Frigoribacterium sp. strain D8. Predicted proteins are marked with green rectangles. Conversion of cellulose to d‐glucose: EC 3.2.1.4.—endoglucanase; EC 3.2.1.21.—1,4‐β‐d‐glucan glucohydrolase/cellobiose glucohydrolase. Conversion of starch to d‐glucose: EC 2.4.1.64—α,α‐trehalose: orthophosphate β‐d‐glucosyltransferase; EC 3.2.1.1—α‐amylase/maltodextrin maltohydrolase; EC 3.2.1.10—dextrin 6‐α‐d‐glucanohydrolase; EC 3.2.1.133—maltogenic α‐amylase; EC 3.2.1.141–4‐α‐d‐[(1‐>4)‐α‐d‐glucano]trehalose glucanohydrolase; EC 3.2.1.20—maltose glucohydrolase; EC 3.2.1.68—isoamylase; EC 5.4.99.15—maltodextrin 1‐α‐d‐glucosylmutase; EC 5.4.99.16—maltose α‐d‐glucosylmutase. [file EMI4-18-e70283-s001.pdf]

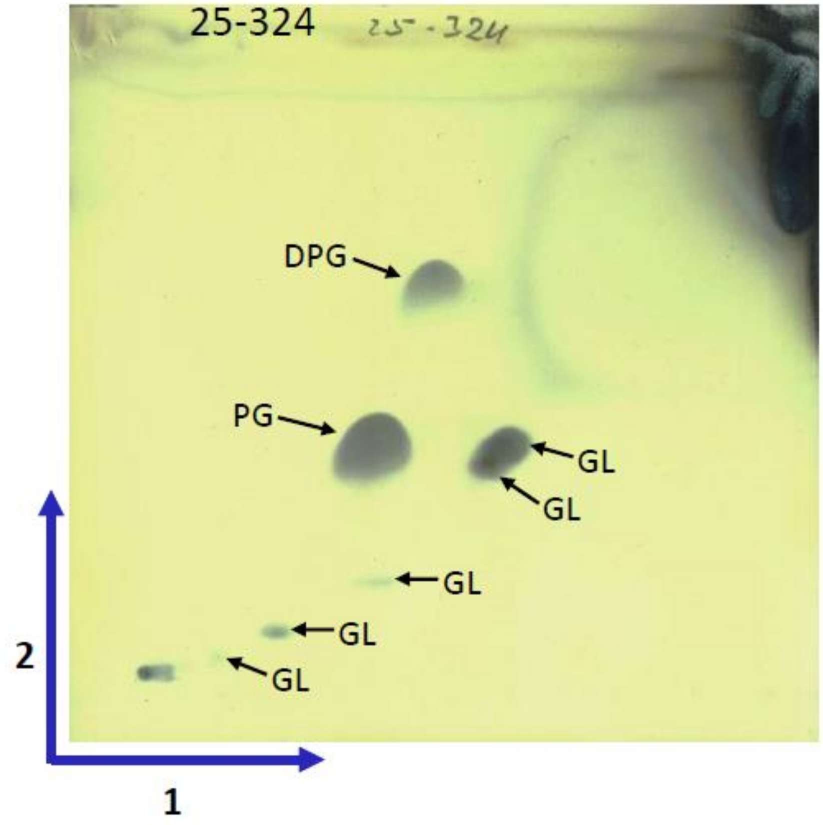

FIGURE S1. The polar lipids of the *Frigoribacterium* sp. strain D8. DPG - diphosphatidylglycerol; PG - phosphatidylglycerol; GL – glycolipid.

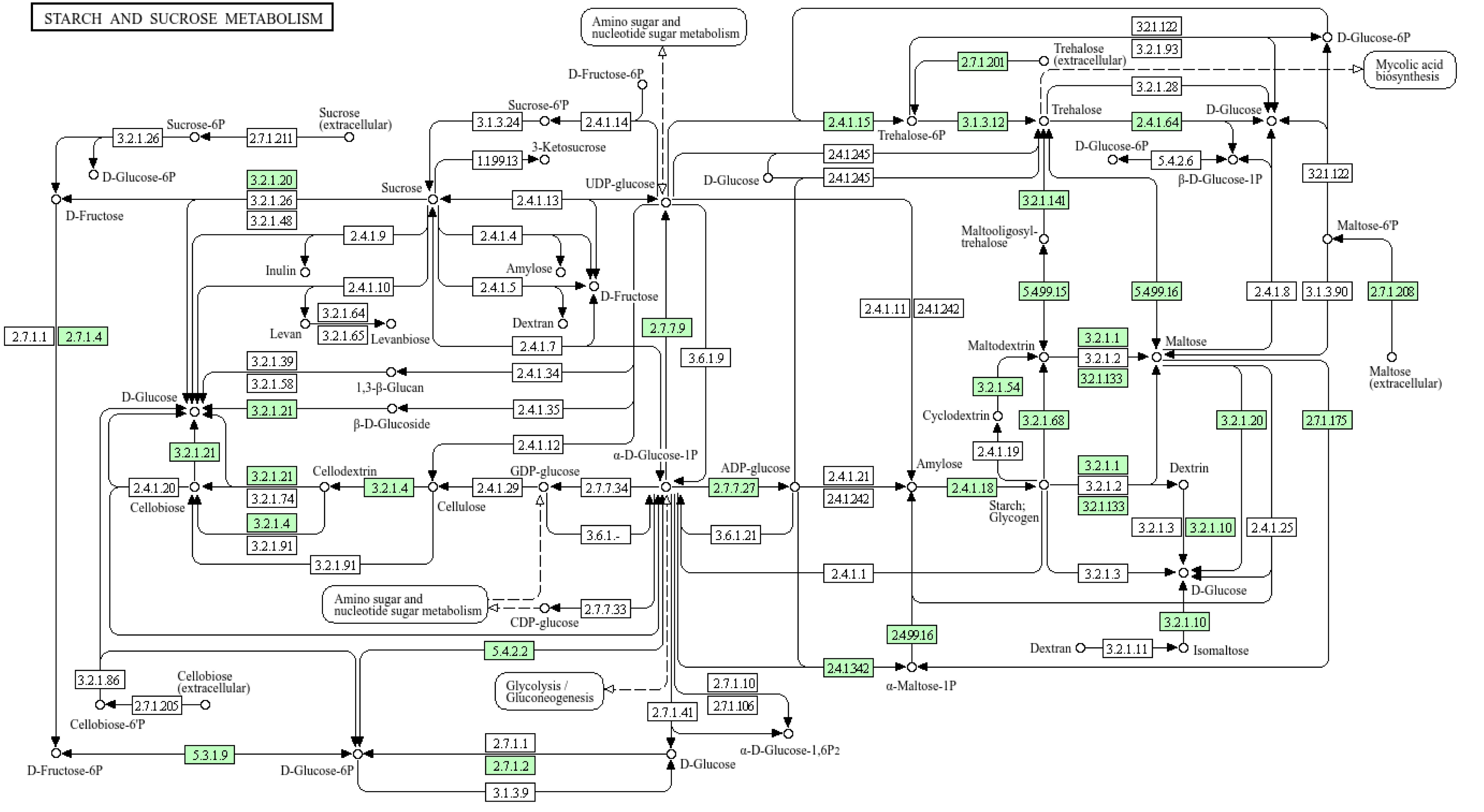

FIGURE S2. *(caption appears on following page)*

FIGURE S2. *(Figure appears on preceding page)* **Starch and cellulose metabolism in the *Frigoribacterium* sp. strain D8.** Predicted proteins are marked with green rectangles. Conversion of cellulose to D-glucose: EC 3.2.1.4. – endoglucanase; EC 3.2.1.21. - 1,4-β-D-glucan glucohydrolase / cellobiose glucohydrolase. Conversion of starch to D-glucose: EC 2.4.1.64 - α,α-trehalose:orthophosphate β-D-glucosyltransferase; EC 3.2.1.1 - α-amylase / maltodextrin maltohydrolase; EC 3.2.1.10 - dextrin 6-α-D-glucanohydrolase; EC 3.2.1.133 - maltogenic α-amylase; EC 3.2.1.141 - 4-α-D-[(1->4)-α-D-glucano]trehalose glucanohydrolase; EC 3.2.1.20 - maltose glucohydrolase; EC 3.2.1.68 – isoamylase; EC 5.4.99.15 - maltodextrin 1-α-D-glucosylmutase; EC 5.4.99.16 - maltose α-D-glucosylmutase.
